# The influence of dopaminergic gene variants on decision making in the ultimatum game

**DOI:** 10.3389/fnhum.2013.00242

**Published:** 2013-06-04

**Authors:** Martin Reuter, Andrea Felten, Sabrina Penz, Anna Mainzer, Sebastian Markett, Christian Montag

**Affiliations:** ^1^Department of Psychology, University of BonnBonn, Germany; ^2^Center for Economics and Neuroscience, University of BonnBonn, Germany

**Keywords:** decision making, ultimatum game, dopamine, DRD2, DRD4, gene, pro-social-behavior, neuroeconomics

## Abstract

One of the most prominent paradigms in neuroeconomics is the ultimatum game (UG) that provides a framework for the study of pro-social behavior in two players interacting anonymously with each other: Player 1 has to split an endowment with player 2. Player 2 can either accept or reject the offer from player 1. If player 2 accepts the offer then the money is split as proposed by player 1. In case of rejection both players get nothing. Until now only one twin study investigated the heritability of the behavior in the UG. Results indicated a strong heritability for the decision behavior of player 2 whereas no genetic influence on player 1 behavior could be detected. Further studies are mandatory to validate these heritability estimates. However, a first candidate polymorphism, the DRD4 exon III, constituting the biological basis of the heritability in the responder behavior has already been identified in a Chinese sample (Zhong et al., 2010). Until now genetic studies in Caucasians on the UG are lacking. The present study wants to fill this gap by investigating the UG in a healthy German sample. Moreover, we intend to find candidate genes that are associated with the first-mover-behavior. In a sample of *N* = 130 healthy participants an online version of the UG was conducted and polymorphisms of the dopamine D2 receptor gene (DRD2) and the DRD4 exon III VNTR were genotyped. We could confirm the DRD4 exon III effect on the responder behavior and the absence of an effect on the proposer behavior reported before. In line with Zhong et al. (2010) carriers of the 4/4 genotype showed a significant higher minimal acceptable offer (*p* = 0.023) than subjects with any other genotype. Furthermore, a DRD2-haplotype-block containing the single nucleotide polymorphisms rs1800497 and rs2283265 was significantly associated with the amount player1 offered (*p* = 0.005) but not with the decision of player 2. Results support the importance of the dopaminergic system for pro-social behavior.

## Introduction

Every day we take numerous decisions that influence our current behavior and often even our future. Sometimes we are confronted to choose between two alternatives that come in the form of an ultimatum: Another person or party makes us an offer that we have either to accept in its present form or that we can reject. In any case we have to bear the consequences. Such a situation can be characterized as a “take it or leave it” situation. Behavioral economists have developed paradigms (so called games) that allow the investigation of human decision making under experimentally controlled conditions [for an overview see Camerer (2003)]. One of these paradigms, the ultimatum game (UG), exactly reflects the above mentioned “take it or leave it” situation. Out of a pool of participants two anonymous players interact in a dyadic situation. One of the players is randomly assigned the role of the first and the other the role of the second mover. Player 1 (also referred to as first mover or proposer) has to split an endowment (e.g., 10 €) between himself and player 2 (also referred to as second mover or responder). If player 2 accepts the offer, the pie is distributed according to player 1's suggestion. If player 2 rejects the offer, both players receive nothing (0 €). According to the assumptions of the economic *Game Theory* player 2 should accept all offers greater than 0 € (Camerer, 1997). However, empirical data from numerous studies contradict this prediction. About half of the offers are declined if they are lower than 30% of the pie (Roth, 1995), i.e., people prefer to dispense with something altogether than being satisfied with at least a small proportion of the pie. Rejecting an unfair offer at one's own cost in order to punish the proposer is not in line with economists' view on man as *homo economicus*. It is stated that the *homo economicus* makes decisions guided by self-interest (maximization of personal benefit) and that his decisions are completely rational (Persky, 1995). Instead of being rational the responder's action is interpreted as a measure of fairness preference. In contrast, the proposer's offer is interpreted as a mixture between fairness preference (to be a social human being that is able to take the perspective of the responder) and strategic considerations (maximize the own profit while minimizing the risk of being punished for an unfair offer). Empirical data on the first-mover-behavior shows that the average offer ranges between 40 and 50% (Camerer and Thaler, 1995) indicating that people are mostly fair. The UG has been successfully applied in cross-cultural studies revealing variance in the behavioral data across countries and ethnicities (Henrich et al., 2001). Whether an act is judged as fair or not is doubtlessly influenced by environmental effects (e.g., upbringing, moral standards, culture) but also genetic factors are conceivable for the following reasons: (a) empirical data in the UG show variability indicating individual differences, (b) ethnical differences in behavior could be caused by differences in allele frequencies across ethnicities, (c) fairness is a facet of pro-social personality traits (e.g., cooperativeness) and traits are highly heritable (up to 50%, Bouchard et al., 1990). Indeed first evidence based on a Swedish twin study showed that more than 40% of the variation in subjects' rejection behavior in the UG is explained by additive genetic effects (Wallace et al., 2007). These data underline that the etiology of fairness preferences has a strong genetic basis. A first study is available now that has identified the DRD4 gene as one out of several potential gene loci that constitute the molecular basis of this heritability (Zhong et al., 2010). The DRD4 gene consists of 3400 base pairs (bp), is located at chromosome 11p15.5, and codes for the dopamine D4 receptor. In exon III of this gene a highly polymorphic variable number of tandem repeats (VNTR) polymorphism has been identified that is characterized by a repetitive sequence of 48 bp (between 2 and 11 repeats) (Van Tol et al., 1992). Three alleles are most common, the 2-repeat, the 4-repeat, and the 7-repeat, whereas the prevalence of the ancestral 4-repeat allele is highest across ethnicities. In Caucasians the 7-repeat is more frequent than the 2-repeat allele, however in Asians the 7-repeat allele is extremely rare and therefore in Eastern populations the 2-repeat allele is the second most common allele. Besides reported associations between the DRD4 exon III polymorphism and various phenotypes related to decision making behavior like impulsivity, novelty seeking, gambling behavior and attention-deficit hyperactivity disorder (ADHD) the functionality of this polymorphism has been demonstrated (Ebstein et al., 1996; Strobel et al., 1999; Eisenegger et al., 2010; Nikolaidis and Gray, 2010). The VNTR region of the DRD4 gene encodes a portion of the third intracellular loop region of the transcribed receptor protein that spans the nerve cell membrane and mediates interaction with second messenger proteins. The 2-repeat allele shows a 50% reduction in the production of cyclic adenosine monophosphate (cAMP) as compared with the 4-repeat and 7-repeat alleles (Asghari et al., 1995).

Although in the majority of these genetic association studies the 7-repeat allele caused the effects in Caucasian samples, it is the homozygous 4-repeat genotype that turned out to be related to economic decision making: Regarding the UG, Zhong et al. (2010) reported that carriers of the 4/4 genotype stated a 25% higher minimal acceptable offer in the role of the second mover as compared to carriers of the 2/4 and 2/2 genotypes. Notably, these results came from a Chinese sample where the 7-repeat allele is absolutely rare and was therefore not in the focus of our analyses. The authors did not find an association between the DRD4 exon III polymorphism and the UG proposer behavior. This is in line with the fact that there are no heritability estimates for the UG proposer behavior available in the literature until now. Although Zhong et al. reported a significant association between the DRD4 gene and fairness as measured by the UG, the proportion of explained variance is rather small. This is typical for quantitative traits and underlines the necessity to identify further genetic variants influencing the behavior in the UG. In this endeavor we have further focused on the dopaminergic system. Especially the DRD2 receptor gene has been related to various facets of pro-social behaviors like cooperation, attachment style, mentoring, paternal parenting, and positive emotionality to name but a few (Lucht et al., 2006; Reuter et al., 2006; Shanahan et al., 2007; Gillath et al., 2008; Walter et al., 2011). Two polymorphisms for which functionality has been proven are most investigated in genetic association studies the DRD2/ANKK1-Taq Ia (rs1800497) and the DRD2 C957T (rs6277) polymorphism. The DRD2/ANKK1-Taq Ia polymorphism is a restriction fragment polymorphism on chromosome 11 at q22-q23 (Pohjalainen et al., 1998; Reuter et al., 2006). Three genotypes of the dopamine DRD2/ANNK1-Taq Ia locus can be differentiated: The A1A1 genotype (also referred to as TT genotype), the A1A2 genotype (also referred to as TC genotype), and the A2A2 genotype (CC genotype). Due to the small prevalence of the A1A1 genotype (3% of healthy Caucasians), A1A1 and A1A2 subjects are commonly grouped as A1+ subjects, whereas A2A2 subjects are referred to as A1− subjects. The prevalence of at least one A1 allele (A1+ group) leads to up to 30% reduction in D_2_ receptor density (e.g., Pohjalainen et al., 1998). The direct impact of the DRD2/ANKK1-Taq Ia polymorphism on D_2_ receptor density has recently been questioned since this SNP is located <10 kb downstream of the DRD2 gene within a protein-coding region of the adjacent ANKK1 gene (Neville et al., 2004). Zhang et al. (2007) investigated 23 SNPs within the DRD2 gene and found a decreased expression of the short splice variant of the D2 receptor compared to the long splice variant caused by two intronic SNPs (rs2283265 and rs1076560). Interestingly, in the study by Zhang et al. (2007) the minor alleles of the two SNPs show strong linkage disequilibrium with the A1 allele of the DRD2/ANKK1-Taq Ia polymorphism (*D*' = 0.855). These data indicate that due to linkage the DRD2/ANKK1-Taq Ia polymorphism is indeed a marker for dopamine receptor density. The DRD/ANKK1-Taq Ia is the most prominent polymorphism with respect to the DRD2 gene. Mostly the minor A1 allele has been related to problematic or non-normative behaviour (e.g., Shanahan et al., 2007; Gillath et al., 2008).

In sum, the present study wants to (a) replicate the reported association between the DRD4 exon III polymorphism and the responder behavior in the UG reported by Zhong et al. (2010). However, this is more than a replication study since in contrast to Zhong et al. we try to test this association in a Caucasian population where the 7-repeat allele is a common allele in comparison to Asian samples; (b) test other dopaminergic gene variants namely polymorphisms on the DRD2/ANKK1 gene that have been related to decision making or pro-social behaviors. It is expected that these dopaminergic polymorphisms have also an influence on the first-mover-behavior in the UG.

## Methods

### Sample

*N* = 130 healthy subjects who are members of the *Bonn Gene Brain Behavior Project* (BGBBP; a gene data bank established with the aim to investigate the genetic underpinnings of human behavior) participated in the present study. The gender distribution was rather skewed [*n* = 105 females (age: *M* = 23.71, *SD* = 6.78) and *n* = 25 males (age: *M* = 25.32, *SD* = 6.63)] which is not surprising because most participants were psychology students at the University of Bonn and most of the psychology students in Germany are female (about 90%). The participants were not familiar with the UG (mainly 1st or 2nd year students participated). Gender groups did not differ with respect to age [*F*_(1, 129)_ = 1.142, *p* = 0.287]. The study was approved by the local ethics committee of the University of Bonn. All participants were completely debriefed on the aim of the study and the rules of the UG in advance of participation.

### The ultimatum game (UG)

The UG was conducted as an online experiment designed in a way that each participant played the game twice, first in the role of the first mover (splitting an amount of 10 € anonymously between himself and another player) and afterwards in the role of player 2 [declaring which minimum amount of money received from player 1 would be accepted by himself (minimal acceptable offer)]. The proposal in the role of the first mover and the minimal acceptable offer in the role of the second mover could be chosen in steps of 0.50 € ranging from 0 to 10 €. Each participant was informed about the consequences of each possible choice in either role: In the role of the first mover, he was instructed that if he for example chooses to send 4 € to the second mover the payoff will be 6 € for himself and 4 € for the interaction partner. Participants were informed that after the end of the study a lottery takes place that randomly builds couples of two players out of the total sample and assigns each participant his actual role in the game (first or second mover). The payoffs are than calculated based on the players' role (first or second mover) and the decisions they had taken before. The payoffs are actually given to the participants after the whole study was completed. There was no additional payment for participation. We contacted about 300 of the BGBBP of whom 130 provided data sets. The duration of the experiment was about 10 min.

### Extraction of DNA and genotyping

DNA was extracted from buccal cells. Automated purification of genomic DNA was conducted by means of the MagNA Pure® LC system using a commercial extraction kit (MagNA Pure LC DNA isolation kit; Roche Diagnostics, Mannheim, Germany). Genotyping of the three SNPs (rs1800497, rs6277, rs2283265) was performed by real time PCR using fluorescence melting curve detection analysis by means of the Light Cycler System 1.5 (Roche Diagnostics, Mannheim, Germany). The primers and hybridization probes (TIB MOLBIOL, Berlin, Germany) were as follows:

DRD2/ANKK1 Taq Ia (rs1800497):

- Forward primer: 5′-CGGCTGGCCAAGTTGTCTAA-3′
- Reverse primer: 5′-AGCACCTTCCTGAGTGTCATCA-3′
- Anchor hybridization probe: 5′-LCRed640-TGAGGATGGC-TGTGTTGCCCTT-phosphate-3′
- Sensor hybridization probe: 5′-CTGCCTCGACCAGCACT-fluorescein-3′

DRD2 c957t (rs6277):

- Forward primer: 5′-GAACTTGTCCGGCTTTACC-3′
- Reverse primer: 5′-CAATCTTGGGGTGGTCTTT-3′
- Anchor hybridization probe: 5′-LCRed640-CCCCGCCAAACCAGAGAAGAAT-phosphate-3′
- Sensor hybridization probe: 5′-TCCACAGCACTCCCGACA-fluorescein-3′

DRD2 rs2283265:

- Forward primer: 5′-TCTTGGGCTAGACGCAT-3′
- Reverse primer: 5′-GTGGAATCCTCAAGACCACC-3′
- Anchor hybridization probe: 5′-LCRed640-CCTGTTTCCTCATCTGTTAAATGGGAAT-phosphate-3′
- Sensor hybridization probe [T]: 5′-TTAGGCAAGTTTCTTACCTTCTATGA-fluorescein-3′

DRD4 exon III:

The DRD4 exon III VNTR polymorphism was amplified from genomic DNA using polymerase chain reaction (PCR) and the primers 5′-TCCTCCGCTTTGGCGCCTCTTCC′ (forward) and 5′-TGGGGGTTGCAGGGGAGATCCTG-3′ (reverse). In brief, after an initial denaturation for 5 min at 94°C, 38 cycles of denaturing at 94°C for 30 s, annealing at 56°C for 30 s, and extension at 72°C for 1 min were followed by a final extension at 72°C for 4 min. PCR amplification was carried out in a final volume of 20 μl consisting of 50 ng genomic DNA, 0.25 mM of each desoxyribonucleotide, 0.5 μM of sense and antisense primers, 2.5 mM MgCl_2_, 10% DMSO, 2 U of Diamond *Taq* polymerase (Eurogentec) and the enzyme supplier's buffer. Amplification products were analyzed by 1.6% agarose gel electrophoresis. The sizes of the common 2-, 4-, and 7-repeats were 379, 475, and 619 bp, respectively. In *n* = 18 subjects genotyping of DRD4 exon III was not possible due to poor DNA quality. The RT-PCR method used for genotyping of SNPs is more sensitive than conventional PCR used for the VNTR. Therefore, valid data for the DRD4 exon III was only available in *n* = 112 subjects. In line with the study by Zhong et al. (2010) subjects with the 4/4 (*n* = 59) genotype were contrasted with the rest of the sample (*n* = 53). Therefore, the DRD4 genotype factor was entered into an ANOVA model with DRD4 as independent factor comprising two levels (4/4 vs. rest).

### Haplotype analyses

Linkage analyses between SNPs and construction of haplotype blocks were conducted by means of Haploview 3.32 (http://www.broad.mit.edu/mpg/haploview/index.php). Individual haplotypes were calculated with PHASE, version 2.1. PHASE implements a Bayesian statistical method for reconstructing haplotypes from population genotype data. In simulation experiments it turned out that the mean error rate using PHASE was about half that obtained by the EM (expectation–maximization) algorithm (Stephens et al., 2001).

## Results

Descriptive analyses of the UG data showed that the average first-mover-proposals (*M* = 4.23, *SD* = 1.53) and the minimal acceptable offers (second mover) (*M* = 3.95, *SD* = 1.69) were comparable to those reported in numerous other studies (Henrich et al., 2001). There were no gender differences, neither for the first mover [*F*_(1, 129)_ = 0.494, *p* = 0.483] nor for the second-mover-behavior [*F*_(1, 129)_ = 1.632, *p* = 0.204] and therefore gender was not included in the ensuing ANOVA models. It has to be pointed out that the absence of a gender effect may be caused by the small proportion of male subjects in our sample. Due to the homogenous student sample age was also not significantly correlated with the dependent variables. First and second mover behavior was significantly correlated (*r* = 0.349, *p* < 0.0001) as it is the case in all UG studies. This means subjects who make fair offers in the role of the first mover have also higher minimal acceptance thresholds in the role of the second mover. The size of this correlation is invariant across genotype groups.

### Genetic analysis

The observed genotype frequencies for the three SNPs under investigation are all in Hardy-Weinberg-Equilibrium (HWE) and are as follows: DRD2 ANKK1/Taq Ia (rs1800497): A1/A1: *n* = 6, A1/A2: *n* = 37, A2/A2: *n* = 87 (HWE: χ^2^ = 0.629, *df* = 1, *p* = 0.428); DRD2 C957T (rs6277): T/T: *n* = 37, C/T: 61, C/C: *n* = 32 (HWE: χ^2^ = 0.470, *df* = 1, *p* = 0.493); DRD2 rs2283265: G/G: *n* = 99, G/T: *n* = 27, T/T: *n* = 4 (HWE: χ^2^ = 1.532, *df* = 1, *p* = 0.216). The following genotype frequencies—that are also in HWE (χ^2^ = 9.111, *df* = 10, *p* > 0.05)—were observed for the DRD4 exon III 48bp VNTR: 2/2: *n* = 2, 2/4: *n* = 13, 2/7: *n* = 3, 4/4: *n* = 59, 4/7: *n* = 25, 3/4: *n* = 6, 5/7: *n* = 1, 7/7: *n* = 3.

### DRD4 exon III

**Figure 1:**
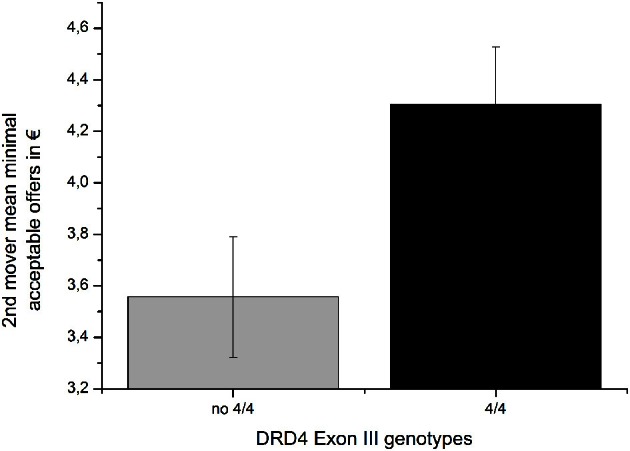
**Second-mover-behavior in the UG minimal acceptable offers in €, mean and SEM**.

We could confirm the DRD4 exon III effect on the responder behavior as reported by Zhong et al. (2010). Carriers of the 4/4 genotype (*M* = 4.305, *SD* = 1.831) stated a significant higher minimal acceptable offer [*F*_(1, 111)_ = 5.329, *p* = 0.023; η^2^ = 0.046] than subjects with any other genotype [*M* = 3.557, *SD* = 1.571; see Table 1 and Figure 1]. With respect to the proposer behavior the 4/4 genotype group (*M* = 4.297, *SD* = 1.529) did not differ significantly from the rest of the sample [*M* = 4.208, *SD* = 1.570; *F*_(1, 111)_ = 0.092, *P* = 0.762; see Table 2] a result that is also in line with the Zhong et al. (2010) study. Due to the fact that most Caucasian association studies on DRD4 exon III concentrated on the 7-repeat allele we in addition compared carriers with at least one 7-repeat allele with participants with no 7-repeat allele. There was neither an effect of the 7-repeat allele on the responder [*F*_(1, 111)_ = 2.595, *p* = 0.110] nor on the proposer behavior [*F*_(1, 111)_ = 0.018, *p* = 0.892].

**Table 1:** **Descriptive statistics (means and standard deviations) for second-mover-decisions in the UG (minimal acceptable offers) dependent on the DRD4 exon III VNTR polymorphism**.

**Alleles**	***n***	***M***	***SD***
2/2	2	4.25	0.25
2/4	13	3.77	0.39
2/7	3	4.00	0.00
4/4	59	4.31	0.24
4/7	25	3.62	0.34
3/4	6	2.92	0.60
5/7	1	5.00	−
7/7	3	2.00	1.26
Total	112	3.95	0.17

**Table 2:** **Descriptive statistics (means and standard deviations) for UG offers (first-mover-proposals) dependent on the DRD4 exon III VNTR polymorphism**.

**Alleles**	***n***	***M***	***SD***
2/2	2	4.75	0.25
2/4	13	4.38	0.18
2/7	3	4.17	0.83
4/4	59	4.30	0.20
4/7	25	4.24	0.39
3/4	6	3.50	0.88
5/7	1	5.00	−
7/7	3	4.00	0.58
Total	112	4.25	0.15

### Haplotype analysis of the DRD2/ANKK1 gene

**Figure 2:**
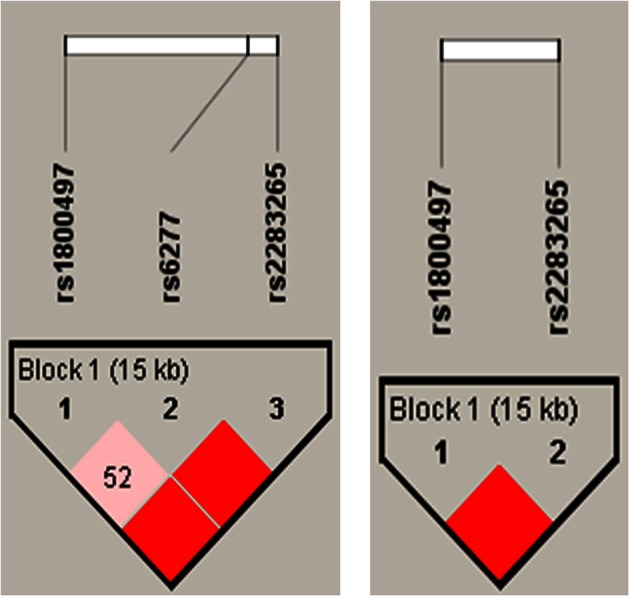
**Results of the DRD2/ANKK1 haplotype analyses. Left panel:** identification of a three SNP haplotype block using the rather liberal *solid spine of LD* method. **Right panel:** identification of a two SNP haplotype block using the more *conservative four gamete rule* method.

Construction of haplotypes revealed a haplotype block encompassing all three DRD2/ ANKK1 SNPs when using the rather liberal *solid spine of LD* method. However, the linkage between DRD2 ANKK1/Taq Ia (rs1800497) and DRD2 C957T was not satisfactory (*D*' = 0.52). The more conservative *four gamete rule* resulted in a two SNP haplotype block with the genetic markers DRD2 ANKK1/Taq Ia and rs2283265 spanning a distance of 15 kb (see Figure 2). Therefore, individual haplotypes were calculated on the basis of this two SNP haplotype block. The empirical haplotype frequencies are presented in Table 3.

**Table 3:** **Empirical haplotype frequencies**.

**Haplotype no.**	**DRD2 ANKK1/Taq Ia rs1800497**	**DRD2 rs2283265**	***n***
1	C	G	211
2	T	G	14
3	T	T	35

**Figure 3:**
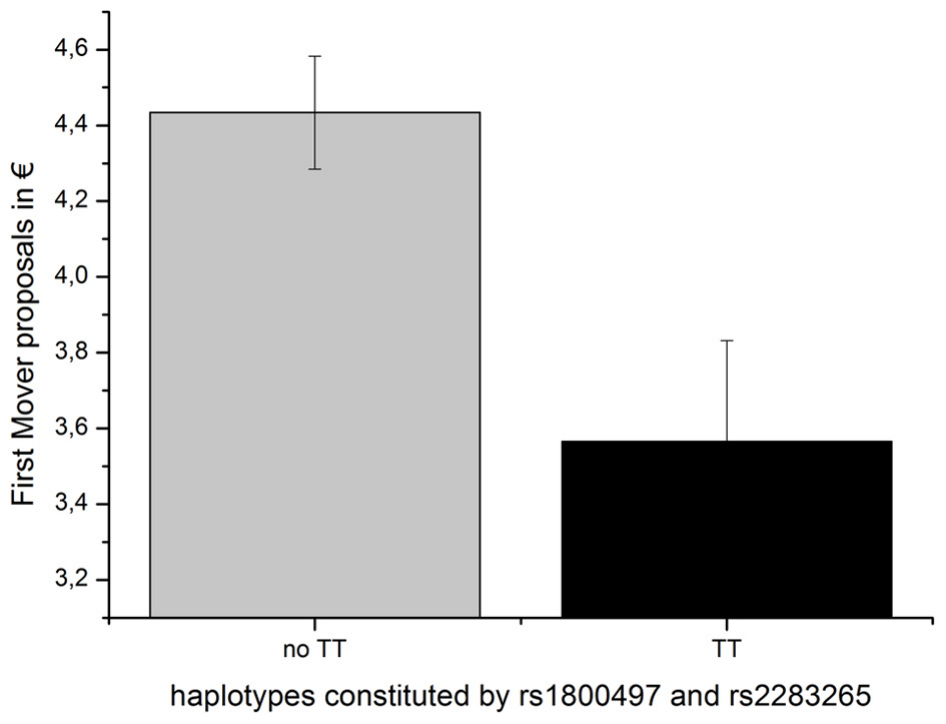
**First-mover-proposal (means and SEMs) dependent on the DRD2 ANKK1 haplotype consisting of the two SNPs rs1800497 and rs 2283265**.

An overall ANOVA model with the DRD2/ANKK1 haplotype as the independent variable and the first-mover-proposal as the dependent variable yielded a trend for a significant effect [*F*_(4, 125)_ = 2.057, *p* = 0.090; η^2^ = 0.062]. An explorative descriptive analysis comparing the mean UG offers dependent on the haplotype genotypes revealed that all participants carrying at least one TT haplotype showed on average lower offers than carriers lacking the TT haplotype completely (see Table 4). Therefore, participants were grouped according to the presence or absence of the TT haplotype (testing those with at least on TT haplotype vs. the rest) in the ensuing analyses. An analysis of variance indicated that the TT group offered significantly less money in the UG (first-mover-proposals) than the no TT group [*F*_(1, 128)_ = 8.102, *p* = 0.005; η^2^ = 0.060; see Figure 3].

**Table 4:** **Descriptive statistics (means and standard deviations) for UG offers (first-mover-proposals) dependent on haplotypes constituted by rs1800497 and rs2283265**.

**Haplotypes**	**Haplotype genotypes**	***n***	***M***	***SD***
11	CG/CG	87	4.44	1.44
12	CG/TG	12	4.38	0.86
13	CG/TT	25	3.50	1.87
23	TG/TT	2	4.00	0.71
33	TT/TT	4	3.75	1.89
Total		130	4.23	1.53

An overall ANOVA model with haplotype as the independent variable and the second-mover-proposal as the dependent variable yielded no significant effect [*F*_(4, 125)_ = 0.510, *p* = 0.729, see Table 5]. Grouping the haplotype groups in the same way as in the analyses of the first-mover-behavior, i.e., comparing the TT haplotype carriers with the other haplotypes, did not result in a significant effect [*F*_(1, 128)_ = 0.119, *p* = 0.731].

**Table 5:** **Descriptive statistics (means and standard deviations) for second mover decisions in the UG (minimal acceptable offers) dependent on haplotypes constituted by rs1800497 and rs2283265**.

**Haplotypes**	**Haplotype genotypes**	***n***	***M***	***SD***
11	CG/CG	87	3.93	0.18
12	CG/TG	12	4.33	0.49
13	CG/TT	25	3.74	0.34
23	TG/TT	2	3.50	1.20
33	TT/TT	4	4.75	0.85
Total		130	4.05	0.32

## Discussion

Recent twin studies have demonstrated that human decision making in economic settings has a strong genetic basis (e.g., Wallace et al., 2007; Cesarini et al., 2008). Studies from molecular genetics trying to identify those gene loci that make up this heritability are rather scarce. With respect to the UG, one of the most prominent games in behavioral economics, Zhong et al. (2010) have reported an association between the 4/4 genotype of the DRD4 exon III polymorphism and the second-mover-behavior. Although this study was conducted in an Asian sample and did not consider the 7-repeat allele that is absolutely rare in the Asian population we were able to replicate this finding in a Caucasian sample where the 7-repeat allele is quite common and therefore included in the analyses. In our sample the carriers of the 4/4 genotype stated a 20% higher minimal acceptable offer than carriers without the 4/4 genotype; in the Asian sample the minimal acceptable offer was 25% higher in the 4/4 genotype group. The responder's action is unequivocally interpreted as a measure of fairness preference that is incompatible with the view on man as *homo economicus*. Interestingly, and complementing our finding Bachner-Melman et al. (2005) found significantly higher self-report scores in altruism in carriers of the DRD4 4-allele and Anacker et al. (2013) in carriers of the 4/4 genotype. In line with the Asian study we could not find an effect of DRD4 exon III on the first-mover-behavior in the UG. Therefore, the present study constitutes an independent replication of the first molecular genetic study on UG behavior—this time in a Caucasian sample. Therefore, the hypothesis could be put forward that the DRD4 effect seems to be invariant across ethnicities. In line with a meta-analysis by Camerer and Thaler (1995) the first mover offer in the present study was 42% on average, although our data were collected via the internet. This result indicates that UG data collected via the internet is comparable to data from experimental sessions in the laboratory.

Interestingly we could find an association between a haplotype block, spanning 15 kb of the DRD2/ANKK1 region consisting of the rs18000497 and rs2283265 SNPs, and the first mover offer in the UG. Carriers of at least one TT haplotype offered significantly less money in the UG (first-mover-proposals) than carriers without a TT haplotype. The proposer's offer is interpreted as a mixture between fairness preference (to be a social human being that is able to take the perspective of the responder) and strategic consideration (maximize the own profit while minimizing the risk of being punished for an unfair offer). The TT haplotype indicates that a subject has at least on one chromosome the minor alleles of both gene variants. Both minor alleles have been associated to lower receptor DRD2 density or decreased relative expression of DRD2s mRNA respectively (Pohjalainen et al., 1998; Zhang et al., 2007). On the other hand the second-mover-behavior was not related to genetic variations in the DRD2/ANKK1 region. Due to the restricted sample size we could not test for interaction effects of the DRD2 and DRD4 variants.

In sum, we find a genetic dissociation between DRD2 (first mover) and DRD4 (second mover) related behavior in the UG that needs further clarification. The neuroanatomical differences in receptor distribution qualify as a valuable starting point for this investigation.

D2 receptors are members of the dopamine receptor G-protein-coupled receptor family that also includes D1, D3, D4, and D5. They are expressed primarily in sub-cortical regions like the nucleus accumbens and caudate putamen where they are involved in the modulation of locomotion, reward, reinforcement, learning, and memory (e.g., Wise, 2004; Klein et al., 2007; Jocham et al., 2009; Frank and Fossella, 2011). Although the DRD4 receptor is also expressed in sub-cortical regions like the amygdala and the midbrain it is also amply located in the frontal cortex (e.g., Oak et al., 2000). The interaction with DRD2 may modulate dopamine- and DA-agonist-induced downstream signaling, i.e., a top-down regulation of emotional processes by central nervous input modulated by DRD4 receptors. First imaging data are available scanning the second movers' brain activity while responding to fair and unfair offers (Sanfey et al., 2003). An increased BOLD response could be detected in response to unfair offers in emotion- (anterior insula) and cognition- (dorsolateral prefrontal cortex) related brain regions. Moreover, Gospic et al. (2011) could demonstrate that also sub-cortical regions, especially the amygdala, are related to the immediate rejection of unfair offers in the UG. These fMRI findings fit perfectly to the behavioral data and the DRD4 gene effects observed in the present study because DRD4 receptors are dominantly expressed in the brain regions triggering the imaging effects. A first fMRI study investigated the brain activity of first movers in the UG (Weiland et al., 2012) and found that fair offers were related to enhanced activity in prefrontal areas, particularly in the subdivisions involved in reward processing and theory of mind. The authors interpreted these findings with the hypothesis that egoistic motives are primarily responsible for fair offers in UG and label this phenomenon as strategic fairness. At first glance, the pronounced role of cognitive aspects in first movers' decision making contradict the DRD2 gene effect reported in the present study because it is assumed to be primarily of sub-cortical nature. However, although strategic, the first-mover decision is not free from affective components, e.g., pity or benevolence for the second mover. Therefore, also sub-cortical effects triggered by sub-cortical DRD2 receptors are likely to influence the proposals in the UG. Nevertheless, it has to be pointed out that the observed gene effects do not allow to directly infer to brain structures related to the UG behavior unless genetic imaging studies have proven such associations.

The strategy to investigate several SNPs on the ANKK1/DRD2 gene simultaneously by means of a haplotype analysis is an elegant method to increase the amount of explained phenotypic variance. Ensuing univariate analyses help to identify the gene variant that drives the genetic effect. In the present study the effect of rs2283265 [*F*_(1, 128)_ = 8.10, *p* = 0.002] on the first-mover-behavior was stronger than that of rs1800497 (DRD2 Taq Ia) [*F*_(1, 128)_ = 5.44, *p* = 0.021] indicating that the association between rs1800497 is probably attributable to a strong linkage with the putative causal effect of rs2283265.

Whereas the second-mover-behavior in the UG is unequivocally interpreted as a measure of fairness preference, the first-mover-proposal is a heterogeneous mixture between strategic considerations and pro-social perspective taking. Future experimental designs investigating the UG and the related dictator game in a within-subject design could disentangle these two components. In contrast to the payoff in the UG that is dependent on the acceptance/rejection of the first mover's proposal by the second mover, the first mover in the dictator game makes a proposal that is implemented independently of the second mover. The identification of distinct gene loci related to the proposals in the UG and the dictator game would contribute to clarify this issue. A shortcoming of the present study is the skewed gender distribution. Although we did not find gender effects there is work pointing to the relevance of gender differences and of sex hormone genes for decision making in the UG (Chew et al., 2013).

In sum, the present study corroborates previous findings demonstrating an influence of the DRD4 exon III polymorphism on second-mover-behavior in the UG and identifies a DRD2/ANKK1 haplotype associated with strategic fairness of the first-mover-decision. Results underline the importance of cortical and sub-cortical dopaminergic activity on social decision making. Although the genetic effects explain at the maximum 6% of the variance, such an effect size is rather large for genetic association studies. Nevertheless, it is necessary to search for additional gene variants that are also related to the decision behavior in the UG and human social behavior in general [for a comprehensive review see Ebstein et al. (2010)].

### Conflict of interest statement

The authors declare that the research was conducted in the absence of any commercial or financial relationships that could be construed as a potential conflict of interest.
